# An extension of the Alma-Ata vision for primary health care in light of twenty-first century evidence and realities

**DOI:** 10.12688/gatesopenres.12848.1

**Published:** 2018-12-14

**Authors:** Henry B. Perry

**Affiliations:** 1 Department of International Health, Johns Hopkins Bloomberg School of Public Health, Baltimore, MD, 21205, USA

**Keywords:** primary heath care, community health, community-based primary health care

## Abstract

This paper builds upon and extends the definition of primary health care in the 1978 Declaration of Alma-Ata. The definition proposes a stronger role for community-based delivery of services and community mobilization, participation and empowerment. It calls for a stronger integration with vertical, disease-specific programs. And, finally, it calls for a strong role for certain curative services (including basic and essential surgery) that many today would not consider as part of primary health care. There is growing evidence that communities can and should play a stronger role than has traditionally been the case, that community-level workers who are properly trained and supported can provide effective services outside of health facilities, and that primary health centers staffed with non-specialist physicians and even non-physician clinicians can perform many of the lower-level inpatient services now performed at first-level referral hospitals.

An approach to primary health care that is appropriate to the local context and that merges local epidemiological priorities with the communities' perceived priorities will make it possible to engage communities as partners. Currently, essential and basic health care services are available to only one-half of the world’s population. The full development of primary health care as envisioned here will accelerate progress in achieving Health for All as envisioned at the International Conference on Primary Health Care in 1978.

## Introduction

Primary health care in the context of global health and the reality of underserved populations in low-income countries, as opposed to primary medical care in the context of well-developed health systems of high-income countries ^1^, addresses practical approaches to the provision of basic ambulatory health services. Thus, instead of focusing on curative care at health facilities by clinicians, the global context of primary health care builds on the definition of primary health care as defined in the Declaration of Alma-Ata at the International Conference on Primary Health Care in Alma-Ata (now Almaty), Kazakhstan. This paper proposed an expanded definition of primary health care that builds on this original Alma-Ata definition in light of current evidence and current realities.

 The World Health Organization reports that one-half of the world’s population does not have access to essential and basic health services ^2^. Progress in improving the health and nutritional status of the poorest segments of many low-income countries continues to lag behind that of the rest of the world; only 4 of the 74 countries with 97% of the world’s maternal and child deaths actually met both Millennium Development Goals for reduction in child and maternal mortality ^3^. The best single indicator we have of the quality and coverage of primary health care services in a low-income country is the mortality rate of children younger than 5 years of age. Second, the population coverage of the most basic and essential services that fall within the realm of primary health care among mothers and children in low-income populations remains surprisingly low: 60% or less for 13 out of 21 interventions and 40% or less for 6 of 21 interventions ^3^. Basic health-promoting behaviors such as exclusive breastfeeding and hand washing, which we know can be effectively promoted through community-based primary health care programs, are still not the norm. Third, the limits of the decades-long emphasis on specific disease-control programs and selective top-down programs are being increasingly recognized. The lack of funding for, and emphasis on, health systems strengthening and, in particular, the lack of emphasis on strengthening of primary health care programs and the community health service delivery component of primary health care, are becoming recognized. Fourthly, health promotion, community participation, community mobilization, and community empowerment are now part of the domain of primary health care and are recognized as essential for improving the health of impoverished populations, for increasing the coverage of basic and essential services, and for adopting healthy behaviors ^4^. Fifthly, with the emergence of chronic diseases as the major global disease burden of the future and the need for persons with chronic diseases to have access to health services, the need for a functioning primary health care system is becoming ever more obvious. Sixthly, and perhaps most importantly, 1 billion people have never seen a health provider ^5^.

The desire—and right—to live a long, healthy and productive life is one of the most fundamental of human hopes and aspirations, and one of the most important functions that societies strive to fulfil. Without the full development of primary health care, Health for All cannot be achieved.

## An extension of the Alma-Ata definition of primary health care for underserved populations in light of 21 ^st^ evidence and realities

I propose here a definition of primary health care that builds on the historical tradition of global primary health care, but at the same time pushes the borders of the definition in three directions: (1) a stronger role for community-based delivery of services and community mobilization/participation/ empowerment, (2) a stronger role for disease prevention with links to vertical programs, and (3) a stronger role for certain curative services that many today would not consider as part of primary health care.

Primary health care consists of those services that people seek and that providers (individual and organizational) deliver to protect health and to treat basic and uncomplicated illness, disease and injuries—especially those that are public health priorities in terms of disease burden that can be alleviated through cost-effective and affordable interventions and programs. These services include those that can be provided in communities outside of facilities by community-based workers, as well as services provided at facilities by frontline health workers (including auxiliary nurses, graduate nurses, and physicians) without advanced specialized training and without expensive diagnostic and laboratory support. The presence of a basic laboratory, other diagnostic equipment, including basic x-ray and ultrasound capability, and an operating room is appropriate for a primary health care center along with the staff and support required to utilize them.

This definition implies that low-cost diagnostic and laboratory support would be available at a health center. Given the fact that technology is evolving rapidly, some of this support available today would have been considered advanced 20 years ago, and what is considered advanced today may be low-cost and usable in primary health care centers in 20 years into the future. Furthermore, what is considered to be “low-cost” at any given point in time will vary from one setting to another. X-ray capabilities and an operating room to perform certain uncomplicated surgical procedures were in fact included in 1920 as elements of primary health centers proposed by the Health Survey and Development Committee led by Lord Dawson of Penn in 1920 in what is known today as the Dawson Report ^6^, which contains the first known use of the term “primary health center.”

Primary health care is composed of three types of activities: disease-oriented primary health care, services-oriented primary health care, and community-oriented primary health care. Disease-oriented primary health care consists of local efforts to control diseases that constitute a significant health burden in the population and for which disease-control interventions exist. While these activities normally have strong technical and funding support from higher levels of government, they are in fact mostly carried out in the community and should be integrated with the primary health care system. Services-oriented primary health care consists of efforts to extend basic personal health care services to the entire population. Community-oriented primary health care consists of efforts to work in partnership with communities to improve their health. These domains are not mutually exclusive and overlap in many instances.

Each of these three types of primary health care is equally important, and together they function like the three legs of a stool on which the “seat” of primary health care rests. If one leg is poorly developed, the primary health care system is sub-optimally constructed to reach its full potential. For the most underserved populations in low-income countries, disease-oriented primary health care has been the strongest leg of this stool by far, with services-oriented primary health care receiving less attention and community-oriented primary health care receiving far less attention than the other two legs.

Box 1 lists the components that make up an ideal primary health care system for underserved populations in low-income countries.

Box 1. Categories of primary health care for underserved populations in low-income countries*

• Disease prevention

• Screening for priority infectious diseases and other priority health-related conditions

• Treatmenf of priority infections diseases (HIV/AIDS, tuberculosis, and malaria)

• Health promotion

• Basic and essential services for women, mothers and children.

• Appropriate treatment of common diseases and injuries

• Basic and essential surgical care

• Community engagement

• Attention to the social determinants of ill health (illiteracy and lack of education, lack of an adequate food supply and adequate housing).

*Some, but not all of these were mentioned specifically in the Declaration of Alma-Ata

Disease prevention: Education about prevailing preventable health problems and methods of preventing and controlling them; provision of preventive services such as immunizations against major infectious diseases, provision of clean water and sanitation, micronutrient supplementation, distribution of insecticide-treated bed nets and intermittent preventive treatment of malaria in pregnant women and children in malaria-endemic areas.

Screening for priority infectious diseases and other health-related conditions: HIV infection, cervical and breast cancer, under- and over-nutrition.

Treatment of priority infectious diseases: HIV/AIDS, tuberculosis and malaria.

Health promotion: Education about behaviors and health care utilization that promote good health, including hand washing, clean water, sanitation and household cleanliness as well as dental hygiene, menstrual hygiene, safe sex, and birth spacing; assurance of good nutrition, especially in mothers and infants; promotion of smoking cessation, weight reduction for those who are obese, physical activity for those who are sedentary, and moderation of alcohol intake; and awareness of warning signs of serious illness for which medical care should be sought.

Basic and essential services for women, mothers and children: Provision of first-line family planning services (birth control pills, condoms, injectable contraceptives, subcutaneous contraceptive implants; detection and treatment of reproductive tract infections; screening and providing initial treatment for breast and cervical cancer; antenatal care; education about recognition of, prevention of, and need to obtain care for complications of pregnancy and childbirth, proper care of newborns and young children, and warning signs of newborn and childhood illness for which attention from properly trained health care providers should be sought; provision of safe and hygienic delivery and treatment of common obstetrical complications, including infection, retained products of conception or retained placenta, management of obstructed labor, prevention and treatment of post-partum hemorrhage, treatment of pre-eclampsia and eclampsia, and management of abnormal fetal presentation/prolonged labor; prevention, detection and treatment of pre-term birth, of birth asphyxia, and of neonatal infection; prevention, detection and treatment of childhood undernutrition, pneumonia, and diarrhea; and recognition of serious neonatal and childhood illness. Management of obstetrical complications requires the capacity for giving parenteral medications and blood transfusions and performing evacuation of the uterus and cesarean section, all of which can be performed in an adequately equipped primary health care center staffed with health personnel who do not have highly specialized training.

Appropriate treatment of common diseases and injuries: Provision of first aid, acute illness management, chronic disease management, pain management, and mental illness care with the full complement of essential medicines; treatment of eye and skin infections, acute respiratory infection/uncomplicated pneumonia; and recognition and referral of life-threatening conditions.

Basic and essential surgical care: “First-line” surgical care (repair of uncomplicated hernias, initial surgical management of the acute surgical abdomen (appendectomy, management of intestinal perforation or uncomplicated intestinal obstruction, and so forth), circumcision, and cataract surgery, all of which can be performed by non-specialist clinicians in primary health centers that can also serve as a first-line basic hospital. Other surgical procedures can be performed outside of hospitals under local anesthesia by appropriately trained and supervised non-physician surgeons during “campaigns”, in which large numbers of patients come to specially formed teams to receive these services. Examples of such procedures include tubal ligation, vasectomy, circumcision, cataract extraction, and herniorraphy) in addition to burn, wound, and fracture management; and cesarean section.

Assistance in the rehabilitation of people with long-term disabilities: Helping people with physical and mental disabilities to become as functional as possible in the community.

Community engagement: Engaging the community in understanding and addressing their health priorities and the determinants of these problems through community-based workers, participatory women’s groups, and participation in community oversight and advocacy activities; and monitoring of local population health status through registration of vital events and periodic population-based health surveys.

Attention to the social determinants of ill health: Literacy and basic education, promotion of adequate food supply and appropriate dietary intake, and provision of adequate housing.

## Further elaboration on this extended definition of primary health care

This updated definition of primary health care seeks to give emphasis to the importance of extending services into the community and households and helping communities to engage in activities that will improve health. Including the term “care” in the phrase primary health care conveys, unfortunately, the notion that a service is being provided to treat an illness or that a service is being provided to prevent an illness. However, as stated previously, the concept of health promotion to adopt healthy household behaviors and appropriate utilization of health services is now fully embedded in the emerging new vision of primary health care.

Disease prevention at the local level, promotion of healthy behaviors, and timely utilization of priority health services fall within the domain of primary health care. Primary health care includes the provision of health-related services provided to persons in their homes, communities, and at ambulatory health facilities by primary health care practitioners. Primary health care is increasingly provided by health teams. I am also proposing that the concept of primary health care should include first-line, lower-level inpatient care that can be provided at a health center, including basic and essential surgical services such as caesarean sections.

In its conceptual and functional sense, primary health care is not tied to any specific type of health facility or to any type of health care provider. Mothers are primary health care providers in their homes for their children – probably the most important of all primary health care providers in impoverished populations. They prevent and treat more illnesses than the formal health system does. In addition to mothers, primary health care is provided by community health workers (CHWs), auxiliaries, nurses, physicians, traditional healers, drug sellers, and informal or non-formally trained practitioners.

In its narrowest traditional sense, primary health care is simply ambulatory health services that are provided by frontline health workers, both formal and informal. Thus, in the narrow traditional sense, primary health care is what I am calling services-oriented primary health care. But this concept of primary health care fails to include disease-oriented primary health care and community-oriented primary health care.

The 1978 International Conference on Primary Health Care and its Declaration of Alma-Ata served as the much-needed catalyst to shift the paradigm of primary health care away from the Western notion of primary medical care (with its focus on the provision of ambulatory health services provided by non-specialist physicians or mid-level clinicians such as nurse practitioners and physician assistants at health facilities such as medical offices, health centers, or outpatient clinics of hospitals) to the notion of “essential care” that could be provided in settings where such infrastructure is non-existent. The Declaration states that primary health care is:

[E]ssential health care based on practical, scientifically sound and socially acceptable methods and technology made universally accessible to individuals and families in the community through their full participation and at a cost that the community and country can afford to maintain at every stage of their development in the spirit of self-reliance and self-determination ^7^.

The 1978 International Conference on Primary Health Care was the largest and most representative global health conference that had been held up to that time, with representatives of 134 governments and 67 international organizations ^8^. It is, then, a definition that had broad legitimacy when issued and has consistently been affirmed since as the “gold standard” for primary health care. It includes the three types of primary health care I have specified. The Conference also called for the achievement of Health for All through primary health care by the year 2000—a goal obviously not yet met, but which will remain with us for at least most of the 21 ^st^ century before it is achieved.

Primary health care responds to patient-defined needs for illness care and also proactively addresses local epidemiological priorities in the community. Primary health care is therefore important for improving the health status of the community. Local epidemiological priorities are the most frequent, serious, readily preventable or treatable conditions in the community. Since epidemiological priorities vary from one locality to another, they are best determined from locally acquired surveillance data. Admittedly, this is not readily accomplished, but epidemographic surveillance has long been proposed ^9^ and is feasible to incorporate into primary health care systems that utilize community-based workers to visit all households on a regular basis. And, in fact, it provides an opportunity for linking civil registration of vital events with primary health care services and thus improving registration of births and deaths in low-income countries, a recognized global priority ^10^. Addressing epidemiological priorities also involves addressing the basic underlying physical and social determinants of ill health—access to safe water, sanitation, good nutrition, adequate housing, and basic education.

There is no general global standard of what constitutes a primary health care center in low-income settings. At present, many of these health centers have inpatient beds and provide inpatient care but do not commonly have the capability to provide—at least for now—the basic laboratory and diagnostic procedures or the basic surgical procedures that should become standard for primary health care programs throughout the world, as will be discussed later.

Households and communities are resources, not just targets, for implementing primary health care. Because of the scarcity and expense of physical health facilities in low-income countries and the higher-level professional personnel who staff them, much of primary health care needs to be provided in homes and communities by community-based staff who are properly trained and supervised in implementing evidence-based interventions and carrying out procedures that have been scientifically validated for provision by lower-level health workers, including illiterate CHWs in populations with high levels of illiteracy.

Health teams led by higher-level health personnel are necessary to train and support CHWs, and a strong system of logistical support for supplies, medicines and commodities is also required. Primary health care is that element of the health systems that can make the greatest contribution to improving population health. As such, it should be the foundation of health systems, particularly in resource-constrained settings. Strengthening the provision of primary health care services should be the first health priority of governments.

## How can a core package of PHC services that are appropriate for the local context be defined?

The updated definition of primary health care provided at the outset contains a full expression of what services need to be provided to disadvantaged populations of low-income countries (and of course to all people everywhere). Out of this full set of services, what portion might be identified as a core set of priority services to be provided?

Efforts to do this have usually used cost-effectiveness criteria for population health benefits, but this approach ignores the obvious fact that people have always needed, and will always need, curative medical services for symptomatic conditions, whether or not they have significance for mortality or serious disability. Urinary infections, arthritis, wrist fractures, toothaches and eye infections, to mention only a few examples, even if they have a low likelihood of causing death or long-term serious disability, will produce a desire for treatment from some kind of primary health care provider because of the symptoms they produce. So, a core set of PHC services needs to include curative care along with services that have demonstrable benefit for reduce the risk of death.

How might one go about defining such a core package? To my mind, the most logical way to go about this is through the application of the principles of what are now called the census-based, impact-oriented approach ^11^. This involves a health program developing a partnership with a population, using local surveillance carried out by routine home visits to define epidemiological priorities (the most frequent, serious, readily preventable or treatable conditions in the population) and to help the communities in the population identify what they consider their health priorities to be (and in low-income settings the community’s health priorities often revolve around improving curative care services). Then, with the available resources (financial, infrastructure, and human), develop a plan that addresses program priorities, which are a combination of epidemiological and community-defined priorities. Thus, the actual content of the services would vary from place to place and over time, depending on the local situation. This approach represents a paradigm shift from what has been the norm in global health for the past half-century or more, namely top-down vertical approaches with no meaningful engagement of local populations or local data in establishing program priorities ^12^.

## The neglected demand-side approach to strengthening primary health care

The concept of community participation in primary health care is strongly enunciated in the Declaration of Alma-Ata, with its call for the following:

The people have the right and duty to participate individually and collectively in the planning and implementation of their health care….
Primary health care … requires and promotes maximum community and individual self-reliance and participation in the planning, organization, operation and control of primary health care, … and to this end develops through appropriate education the ability of communities to participate.
Primary health care … relies … on health workers … suitably trained socially and technically to work as a health team and to respond to the expressed health needs of the community ^13^.

In the extended definition of primary health care presented earlier in this paper, community-oriented primary health care is one of the three “pillars” (or legs of the stool) of primary health care, and this involves working in partnership with communities to help them improve their health.

## Roles of higher- and lower-level personnel

The updated definition of primary health care proposed here calls for the inclusion of the full spectrum of primary health care providers, from mothers in the home to community health workers, auxiliary workers, nurses, and physicians. In resource-constrained settings and even in resource-rich settings such as the United States, the team approach to provision of health services leads to the best quality of services and to the most cost-efficient use of available resources. Even in the United States, the use of CHWs as members of health teams is growing rapidly ^14, 15^. There is now a vast global evidence base regarding the suitability and advisability of delegating to lesser-trained staff many tasks and responsibilities that were once the sole purview of physicians and graduate nurses ^16^. Although not without controversy still, the weight of the evidence continues to strongly support the full engagement of carefully selected lower-level workers to carry out many well-defined tasks and responsibilities as long as these persons are suitably trained and well-supervised ^17^.

## Remuneration and other incentives for frontline workers

The remuneration of frontline health workers is a current source of controversy, particularly with respect to whether or not it is appropriate for health programs to engage CHWs on a voluntary, non-salaried basis. Although the amounts of money paid to frontline workers are modest, the vast number of these workers means that the implications of payment policies for CHWs are substantial, especially when the funding comes from a central government source. On the one hand, there is the frequently made charge that engaging CHWs without remuneration is unjust exploitation. Nonetheless, field experiences have demonstrated that communities commonly have people who are eager to serve their neighbors on a voluntary basis. If funding scarcity were not a problem, then payment would of course be desirable. However, there are examples of CHW programs that made initial commitments to pay their workers but then could not maintain that commitment, leading to a crumbling of the program ^a^. If the creation of an expectation for a salary is established and then the program cannot sustain that expectation over time, the program is worse off than if it had originally started with volunteer CHWs who had no expectation of a salary.

In NGO child survival programs there are many examples of volunteer CHWs who receive no formal salary but who receive some kind of “incentives,” in the form of special recognition from the community, release from certain community responsibilities, special privileges in accessing health services, and so forth. Many top-down, highly selective vertical programs provide a short-term daily incentive for a short period of time. The most balanced approach to this issue is to not expect from volunteers more than a modest amount of ongoing weekly work (e.g., no more than 4–5 hours per week) and, if on-going financial remuneration is to be provided, to be confident that this support can be maintained on a sustainable basis.

## Impact of global trends on the evolution of primary health care for the disadvantaged in low-income countries

Anticipating the specific influences of global forces and trends on primary health care is, of course, impossible. We can be sure that more and more of the disadvantaged in low-income countries will be living in slums in urban area. We can be sure that technological advances will bring new diagnostic and laboratory tests within the reach of low-cost primary health care programs, and mobile health (mHealth) will make it easier for people to communicate with their health care provider and easier for different members of the health team to communicate among themselves, providing opportunities for improving the quality of care. As the population ages, chronic diseases will increasingly dominate the disease burden. AIDS will have become a chronic disease, and primary health care will be heavily concerned with care of the elderly. Socioeconomic development will lead to higher living standards and higher educational levels, and this will affect the demand for and consumption of primary health care services. No doubt the trend for women’s empowerment will continue, and this will also affect the demand for and consumption of primary health care services.

## Towards a rebirth and revision of primary health care

In recent articles and writings related to primary health care, there has been a persistent theme: the concept of primary health care as articulated at Alma-Ata, Kazakhstan is valid and needs to be maintained. But at the same time there is a broadly held view that we are now at a time for a renewal and revitalization of primary health care and perhaps even for a re-definition of primary health care for the 21 ^st^ century, leading to an even stronger commitment to achieve the vision of Alma-Ata.

In recognition of the 25 ^th^ anniversary of the Alma-Ata conference in 2003, the Pan American Health Organization convened a series of events and dialogues, culminating in a 2007 position paper on renewing primary health care in the Americas ^18^. Their report states that there is “a growing recognition that PHC is an approach to strengthen society’s ability to reduce inequities in health; and a growing consensus that PHC represents a powerful approach to addressing the causes of poor health and inequality” ^18^ [p. 2]. In celebration of the 30 ^th^ anniversary of the Declaration of Alma-Ata, *The Lancet* published a series of papers entitled “Alma-Ata: Rebirth and Revision.” The lead editorial by The Lancet team stated that “The Alma-Ata Declaration revolutionized the world’s interpretation of health. Its message was that inadequate and unequal health care was unacceptable: economically, socially, and politically.” ^19^ Margaret Chan, Director General of the World Health Organization, in her lead editorial to the series, remarked: “With an emphasis on local ownership, primary health care honoured the resilience and ingenuity of the human spirit and made space for solutions created by communities, owned by them, and sustained by them.” ^20^ One of the articles in the series expressed the same idea this way:

The very idea of health for all energised workers and fueled new efforts in many countries to improve service coverage, especially for previously underserved communities. The inherent focus on equity, the necessity of reaching the unreached and involving them not only in the benefits of health care, but more importantly, in the decisions and actions that collectively make health, was at once novel and revolutionary. Thus, the precepts of social justice became an integral part of health planning ^21^ [p. 919].

The Second International Conference on Primary Health Care ^22^, held in October 2018 in Astana, Kazakhstan, to commemorate the 40 ^th^ anniversary of the Declaration of Alma-Ata, and its Declaration of Astana ^23^, reaffirmed the central importance of primary health care for achieving universal health coverage, the health-related Sustainable Development Goals, and Health for All. It is time to ignite a global renaissance in primary health care as the centrepiece of efforts to end preventable child and maternal deaths by the year 2030. Implementing the updated definition of primary health care provided here will contribute to the achievement of the highest attainable standard of health for all people and especially for the “bottom billion” of our global family.

Now is the time for a renewed focus on the principles of primary health care outlined at Alma-Ata in 1978. Unfortunately, Health for All was not achieved by the year 2000 and may not be achieved by the year 2100. But the full implementation of primary health care as proposed here for the disadvantaged in low-income countries will help to achieve this global human aspiration sooner rather than later.

## Data availability

No data are associated with this article.

## Footnotes

^a^This happened in Nepal in the early 1980s, and later these workers, who had become inactive, were recruited back later as Female Community Health Volunteers.
