# Supplementary material for: An extension of the Alma-Ata vision for primary health care in light of twenty-first century evidence and realities
Source: Gates Open Res. 2018 Dec 14;2:70. [Version 1] doi: 10.12688/gatesopenres.12848.1 (PMC6362300; doi:10.12688/gatesopenres.12848.1)
Supplement: Peer review history

# Peer review: Reviewer response for version 1

**DOI:** 10.21956/gatesopenres.13929.r26827

**Published:** 2019-01-07

**Authors:** Peter Byass

**Affiliations:** 1 Department of Epidemiology and Global Health, Umeå University, Umeå, Sweden

This article discusses developments in approaches and definitions to primary health care. I find it difficult to review against the label “research article” as it much more of a narrative review in structure and content, and indeed states explicitly “no data are associated with this article”.

 The author chooses to make a sharp distinction from the outset between primary health care in low-income countries versus primary medical care in high-income countries, which I find somewhat dated and unhelpful. Of course it is true that any country’s primary care and economic development are inextricably interrelated. However, the majority of the 21 ^st^ century world’s population actually lives in middle-income countries, which are not mentioned. Every country’s health system should be providing both preventive and curative care at the primary level, and indeed this is a fundamental vision of WHO’s current push towards “health for all” and “universal health coverage”, which are absolutely not only for low-income countries. However, the global reality is that there is a spectrum of countries on both health care and economic development axes, and it would have been more helpful for this article to have taken this global perspective as its basis, perhaps going on to extract how countries at the lower end of the development spectrum might most appropriately conceptualise primary care.

 The range of different health care personnel that are needed to provide effective primary care in any situation are also rather unhelpfully dichotomised into “higher- and lower-level personnel”. This seems to go against the concept of primary care teams which have long been a feature of many health systems, for example in European countries, and perhaps reflects an over-emphasis on the very hierarchical model of the US health system.

 Nevertheless there are important pointers here to positive ways in which primary care systems might develop in various settings.

 There is a typo in the first major heading, which should read “light of 21st century evidence”.

I have read this submission. I believe that I have an appropriate level of expertise to confirm that it is of an acceptable scientific standard, however I have significant reservations, as outlined above.

# Peer review: Reviewer response for version 1

**DOI:** 10.21956/gatesopenres.13929.r26828

**Published:** 2019-01-04

**Authors:** Wim Van Damme, Juan Antonio Ricarte

**Affiliations:** 1 Department of Public Health, Institute of Tropical Medicine Antwerp, Antwerp, Belgium; 2 UHC Study Group, University of the Philippines, Manila, Philippines

Thank you for the opportunity to review this important article. Primary Health Care (PHC) indeed is, or should be, the foundation of any health system, and it is timely to revisit the definition of PHC in line with the transitions happening in the world over the past 40 years since the Alma-Ata declaration. The paper contributes to a much-needed discussion on the concept and definition of PHC; stressing the absolute primacy of essential services in contrast to the over-dominance of disease control programs and hospital services, which is observed in many countries.

 Since its inception 40 years ago, the Alma-Ata vision for PHC, has often been interpreted in a restrictive way, as Perry rightly explains. But from the onset, there has also been a school of thought on PHC which embraced a comprehensive vision, close to the one proposed in this paper. This has been framed as a clash between “selective PHC & comprehensive PHC”, or as “vertical vs. horizontal approaches”. The paper rightly tries to go beyond this “false dichotomy” and present an inclusive definition of PHC, acknowledging the importance of all components of an effective health system. It also shows that such vision is not only relevant for low- and middle-income countries, but for all societies.

 The paper presents a good rationale for redefining PHC: given that technology is rapidly evolving and has become more accessible even in low-income contexts. It would be nice to give more examples on this (e.g. new low-cost diagnostics, telemedicine, e-health). The paper also presents relevant and concrete examples of how PHC services can be expanded at the local level, and how community participation should be fully part of PHC.

 However, for this review, we also identify some points that could further strengthen the paper, or need some clarification:

- Relation between PHC & Universal Health Coverage (UHC), which has now become the dominant framing of health care;
- Relation between “continuous essential services” and “disease control programs”, pleading for “integration” is the dominant advocacy term, but “integration” can be interpreted in so many different ways that some more specifics are needed to clarify the What? Why? How? Additional explanation and examples might be needed on “strengthening the integration of PHC to vertical programs”. In addition to the examples on incentivizing HRH aligned to vertical programs, some PHC-vertical program linkages (e.g. alignment of resources/technology, referral systems, alignment of priorities) could be discussed.
- A number of terms used in the article don’t necessarily have universal definitions. For example:
  - A “health center” with surgical capabilities in one context, would be called a district hospital or infirmary in a different setting
  - The term “health provider” is used loosely, (i.e. “1 billion people have never seen a health provider” Vs. “Mothers are primary health care providers”).
- Primary health care is not synonymous to health care provided in a primary care facility. In comprehensive definitions of PHC, essential care in primary hospitals, such as C-sections, is part of the PHC spectrum. Perhaps an alternate approach would be to discuss in terms of essential primary health care services.

We look forward to the final publication.

We have read this submission. We believe that we have an appropriate level of expertise to confirm that it is of an acceptable scientific standard.

# Peer review: Reviewer response for version 1

**DOI:** 10.21956/gatesopenres.13929.r26826

**Published:** 2018-12-20

**Authors:** Peter S. Hill

**Affiliations:** 1 School of Public Health, University of Queensland, Brisbane, Qld, Australia

Thank you for the opportunity to review this paper which revisits Primary Health Care (PHC) following the celebration of its 40 years in action. The proposed "expansion" is consistent with the spirit of PHC, in its local application, and the need to re-invest in the framing in dialogue with Universal Health Coverage.

 There are two areas in which the paper could be enhanced: Firstly, in structural clarity. It would be useful to restate the original definition of PHC that the author is working with--there are more than one definition that are in circulation, but with the paper expanding it would be useful to be specific. Secondly, it would be useful to more overtly indicate which statement is the expanded definition. In the introduction there are five "components" that could represent the new definition; in the next section there are three "directions". It would be helpful if the relation between these was stated, and the actual "definition" more clearly marked. I also found the reiteration of "stronger" problematic in that it did not provide defined endpoints in the expanded activities--it becomes difficult to know at what point the new PHC is achieved. The second issue relates to the level of analysis--this is PHC in a centre/services/activities frame--there is also a higher level of systems analysis that could be approached that would locate these within a national (or universal?) frame. In this regard, reference to the WHR 2008 Primary Health Care now more than ever would be useful because of the reforms and reframing of PHC it advocated--and that would link well to this new visioning at a systems level.

 I look forward to the final publication.

I have read this submission. I believe that I have an appropriate level of expertise to confirm that it is of an acceptable scientific standard, however I have significant reservations, as outlined above.
